# QUIPS-based prospective postoperative pain assessment following nephrectomy and partial nephrectomy in robot-assisted, conventional laparoscopic, and open surgical approaches

**DOI:** 10.3389/fsurg.2026.1782987

**Published:** 2026-06-30

**Authors:** Maximilian Müller, Lars Kurch, Philipp Burow, Michael Bucher, Annett Christel, Winfried Meissner, Johannes Dreiling, Lilit Flöther

**Affiliations:** 1 Department of Urology, Sana Kliniken Leipziger Land, Borna, Germany; 2 Department of Anesthesiology and Surgical Intensive Care Medicine, University Hospital Halle (Saale), Martin-Luther University, Halle, Germany; 3 Department of Nuclear Medicine, University Hospital Leipzig, University Leipzig, Leipzig, Germany; 4 Department of Anesthesiology and Intensive Care Medicine, University Hospital Jena, Friedrich Schiller University Jena, Jena, Germany; 5 Department of Palliative Care, University Hospital Jena, Friedrich Schiller University Jena, Jena, Germany

**Keywords:** laparoscopy, nephrectomy, open surgery, partial nephrectomy, postoperative pain, quality improvement in postoperative pain management (QUIPS), robotic surgical procedures

## Abstract

**Background:**

Postoperative pain is one of the most common medical issues following kidney surgery. It has significant impact on morbidity, recovery and patient satisfaction. To date, limited data are available on the relationships between surgical procedures, postoperative pain progression, and quality-of-life parameters.

**Methods:**

116 patients were recorded prospectively after minimally invasive (robot-assisted and conventional laparoscopic) and open surgical kidney procedures. Data collection was standardized as part of the Quality Improvement in Postoperative Pain Management (QUIPS) project. Primary endpoints were postoperative pain intensities (numerical rating scale, NRS) during movement, maximum and minimum pain. Secondary endpoints included need of analgesics, side effects, patient satisfaction, and length of hospital stay.

**Results:**

Both groups demonstrated high postoperative pain intensities. However, patients undergoing minimally invasive procedures presented with lower pain scores compared to those who underwent open surgery (pain on exercise: median 6 [IQR 4–8] vs. 7 [IQR 5–8], *p* = 0.050; maximum pain: median 7 [IQR 5–8] vs. 8 [IQR 6–9.8], *p* = 0.004; minimal pain: median 1 [IQR 0–2] vs. 2 [IQR 0–3]; *p* = 0.030). The need for opioids on the ward was higher after open procedures [25/62 (40.3%) vs. 11/54 (20.4%); *p* = 0.021]. Patient satisfaction scores were high across both groups [minimally invasive: median 8 [IQR 6–10]; open: median 8 [IQR 6–9]; *p* = 0.905]. Robot-assisted procedures were also associated with the lowest postoperative pain scores [pain on exercise: median 5 [IQR 4–7]; maximum pain: median 6 [IQR 5–8]; minimal pain: median 0 [IQR 0–2]] and the shortest hospital stay [median 7 days (IQR 6.8–9)].

**Conclusion:**

Despite advances in pain management, postoperative pain following kidney surgery remains a significant medical issue. Our data suggest that pain management across different surgical modalities may not be homogeneous. Instead, different operative approaches may require procedure-adapted pain management with tailored analgesic escalation strategies to optimize postoperative recovery and patient satisfaction.

## 1 Introduction

Postoperative pain is one of the most common and clinically significant issues following surgical procedures. It is considered a key quality criterion in perioperative care. Although acute pain fulfils an essential protective function, a high level of postoperative pain was shown to be associated with numerous negative consequences, including delayed convalescence, an increased risk of developing chronic pain syndromes, and a significant reduction in quality of life (1, 2). In addition, high pain intensities correlate with an increased complication rate and longer hospital stays. They also have a significant impact on patient satisfaction and the choice of healthcare facility (3, 4).

Despite the availability of effective analgesic strategies, German S3 guidelines, and international recommendations, as Procedure Specific Pain Therapy (PROSPECT), studies show that approximately half of all patients experience postoperative pain requiring treatment (5). The main causes are considered to be deficiencies in the organization of pain therapy, patient education, structured evaluation, and documentation rather than insufficient pharmacotherapy (6–8).

Initiatives to improve the quality of pain management such as the benchmarking project Quality Improvement in Postoperative Pain Management (QUIPS) enable2 standardized, valid, and patient-centered recording of pain-related outcome parameters. Thus, the project represents an effective tool for improving transparency of care and inter-institutional comparability (9, 10). Structured questionnaires are used to systematically collect data on pain intensity, impaired body functions, side effects, and satisfaction with therapy.

Kidney surgery, in particular radical nephrectomies and partial nephrectomies can be performed using open surgery, conventional laparoscopic techniques or robot-assisted techniques. The latter is gaining importance worldwide as it is associated with improved perioperative results with comparable oncological outcomes (11–13).

Nevertheless, there is limited data available on the quality of postoperative pain management depending on the surgical method chosen. In operations with minimal soft tissue damage, including laparoscopic procedures, the intensity of pain might be often underestimated, even though patients frequently experience severe pain. Therefore, it appears that the correlation between the intensity of postoperative pain and the surgical tissue defect is limited.

Decisive influencing factors include ongoing inflammatory processes, injuries to neural structures, and the type of damaged tissue (14–16). However, early studies reported advantages of robot-assisted procedures, which were associated with lower pain levels, better daily functioning, and higher quality of life during the first postoperative month (17).

This study aimed to use QUIPS as an instrument to systematically evaluate the quality of postoperative pain management after minimally invasive (robot-assisted and conventional laparoscopic) and open surgical kidney procedures. Pain-related outcome parameters and relevant process parameters were collected to characterize the current state of perioperative pain management and identify at-risk patient populations.

## 2 Methods

A prospective observational study was conducted at the Department of Urology and the Department of Anesthesiology and Operative Intensive Care Medicine at Halle (Saale) University Hospital between December 2022 and January 2024. Adult patients who underwent nephrectomy or partial nephrectomy with complete demographic and clinical data were included. Patients with missing informed consent, incomplete documentation, limited ability to communicate for pain assessment, or reoperations during the hospital stay were excluded.

Patients were assigned to one of two main groups based on the surgical procedure: minimally invasive (robot-assisted and conventional laparoscopic) and open surgery. The availability of the surgical robot, the surgeon's experience and skill, the size of the expected finding, and the patient's comorbidity were factors influencing the choice of surgical approach. In additional sub-analyses, robot-assisted, conventional laparoscopic and open procedures were considered separately. The Data collection was standardized using QUIPS (German Clinical Trials Register ID: DRKS00006153). A positive vote was obtained from the Ethics Committee of Halle University Hospital (Identification Number: 2021–094).

All patients received general anesthesia with endotracheal intubation. Four patients who underwent open renal surgery additionally received regional anesthesia. Postoperative analgesia in the post-anesthesia care unit (PACU) and on the ward did not follow a standardized protocol but was determined individually by the attending anesthesiologists and urologists. According to clinical standard procedures, in the PACU paracetamol and oxycodone were predominantly administered, whereas metamizole and piritramide were mostly used on the ward. Both non-opioid analgesics and opioids were administered predominantly on a demand-oriented basis, while patient-controlled analgesia and regional anesthesia techniques were employed in only a small number of cases. Analgesic administration was analyzed in a dichotomous manner, capturing only whether each medication had been administered. Detailed numbers are provided in the Supplementary Tables S1–S4. Analgesia documentation from the post-anesthesia care unit was missing for five patients.

Postoperative pain intensity was assessed using the Numerical Rating Scale (NRS, 0–10), where 0 indicates “no pain” and 10 represents the “worst imaginable pain”. Within the QUIPS framework, pain assessments were conducted by trained nursing staff as a single evaluation within the first five postoperative days. Postoperative day 1 served as the primary target time point, which was achieved in the majority of cases. However, in a subset of patients, assessment at the first postoperative day was not feasible due to clinical or staffing constraints, necessitating data collection at a later postoperative day. All assessments were performed under inpatient conditions. Three pain dimensions were recorded: maximum pain, minimum pain, and pain on exercise (e.g., during mobilization or coughing). Pain-related functional impairments, therapy-associated side effects (e.g., nausea, dizziness, fatigue), and patient satisfaction with pain management were also documented using standardized QUIPS questionnaires. Satisfaction was assessed on an 11-point scale (0 = very dissatisfied, 10 = very satisfied). Demographic items (age, gender), comorbidities according to the American Society of Anesthesiologists (ASA) classification and Charlson Comorbidity Index (CCI), scoring systems to assess mortality in surgical patients, surgical parameters (duration in minutes, type of procedure), preoperative pain medication and information on perioperative pain management were recorded. QUIPS outcome parameters included information on pain intensity (stress, maximum and minimum pain intensities), therapy-associated side effects, pain-related functional impairments, and patient satisfaction with pain therapy.

IBM SPSS Statistics software (version 27.0, IBM Corp., Armonk, NY, USA) was used for statistical analysis. Unless otherwise stated, continuous results are presented as median [IQR]. Group differences in categorical data were analyzed using Pearson's chi-square test. The Mann–Whitney-U-test or analysis of variance (ANOVA) was used for continuous, non-normally distributed or heterogeneous data. Subsequently, univariable analysis was performed to identify clinically relevant variables (*p* < 0.1) with a significant impact on the endpoints. Nominally scaled outcome parameters were analyzed using binary logistic regression. Interval-scaled outcome parameters were examined by stepwise linear regression to assess the combined influence of the preselected variables. Results of the linear regression models are reported as regression coefficients with 95% confidence intervals. The results of the logistic regression models are presented as odds ratios with corresponding 95% confidence intervals. Based on these findings, potential risk groups were identified.

The statistical analyses were conducted using an exploratory study approach. Due to the lack of a confirmatory design, no case number calculation was performed. All evaluations, including univariable and multivariable analyses, serve to describe the data and generate hypotheses for future studies. The *p*-values given should be interpreted as exploratory and do not allow for definitive inferential statistical conclusions.

## 3 Results

### 3.1 Patient characteristics

**Figure 1:**
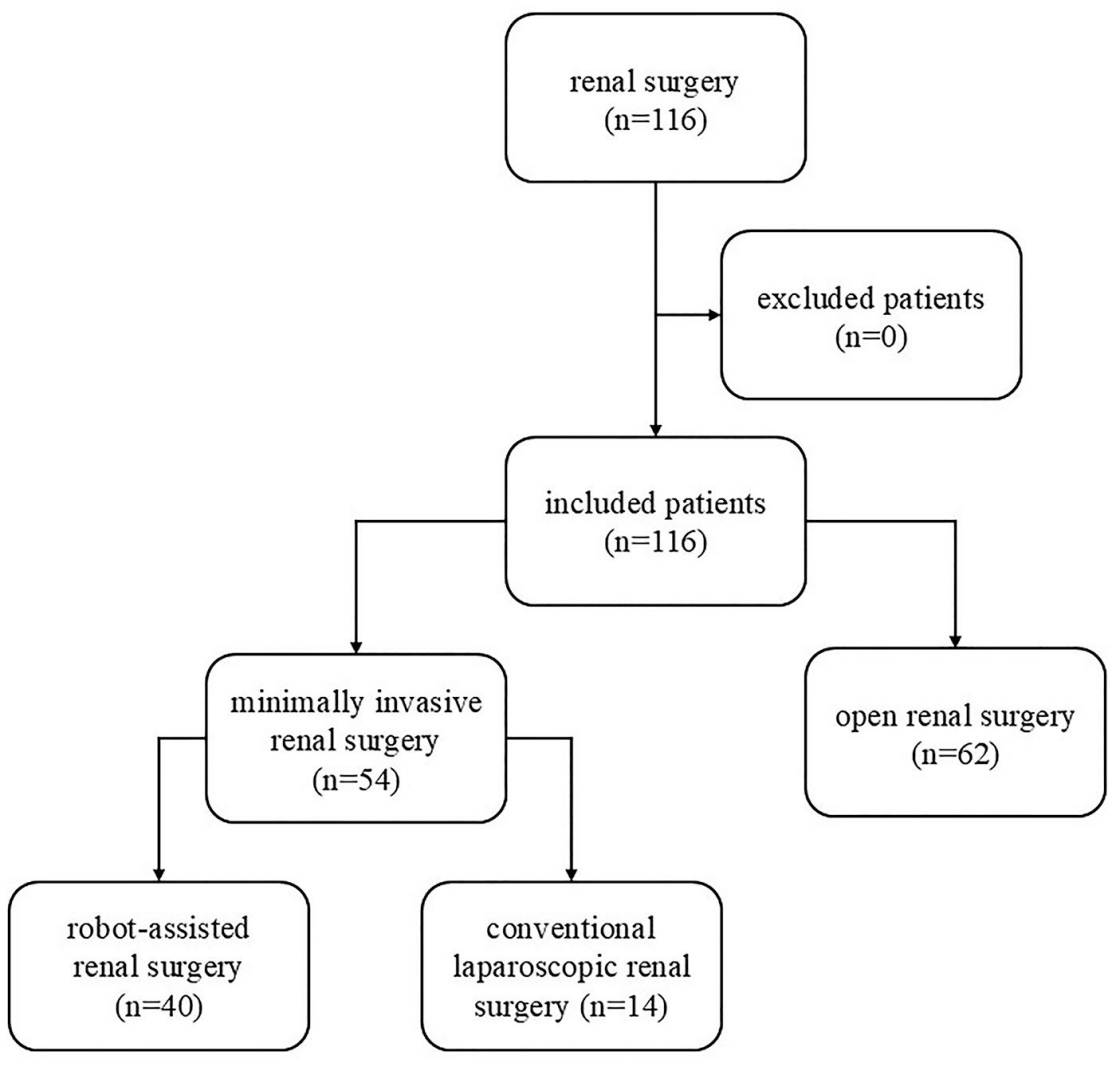
Study flow diagram.

All 116 identified patients who underwent renal surgery met the inclusion criteria. 62 patients underwent open surgery. 54 patients had minimally invasive surgery. The minimally invasive group was further divided into 40 patients who went through robot-assisted surgery and 14 who got conventional laparoscopic surgery (Figure 1).

An overview of the demographic and clinical patient characteristics is shown in Table 1. The mean age was comparable in both groups (62.0 vs. 62.9 years; *p* = 0,696), as was the gender distribution (37.0% vs. 38.7% female; *p* = 0,853). There was also no difference between the two main groups in terms of the presence of preoperative chronic pain and analgesic use. However, there were relevant differences in the preoperative risk profile: Patients who underwent open surgery were more likely to have an ASA status of 3 [41/62 (66.1%) vs. 21/54 (38.9%) for minimally invasive surgery; *p* = 0.006] and had more comorbidities [CCI >0 in 54/62 (87.1%) vs. 39/54 (72.2%); *p* = 0.007]. The extent of resection also differed. While partial nephrectomy was performed more frequently in the minimally invasive group, radical nephrectomies dominated in the open group [53/62 (85.5%) vs. 30/54 (55.6%); *p* < 0.001].

**Table 1:** Patient characteristics.

		surgical technique				
Variable	Subgroup	minimally invasive		open		
		n	%	n	%	*p*-value
sex	female	20	37,0%	24	38,7%	0,853
	male	34	63,0%	38	61,3%	
age	18–20	0	0,0%	0	0,0%	0,696
	21–30	1	1,9%	1	1,6%	
	31–40	4	7,4%	3	4,8%	
	41–50	5	9,3%	0	0,0%	
	51–60	14	25,9%	23	37,1%	
	61–70	14	25,9%	15	24,2%	
	71–80	12	22,2%	15	24,2%	
	81–90	4	7,4%	5	8,1%	
ASA-Classification	1	8	14,8%	2	3,2%	**0,006**
	2	25	46,3%	19	30,6%	
	3	21	38,9%	41	66,1%	
Charlson Comorbidity Index	0	15	27,8%	8	12,9%	**0,007**
	1	2	3,7%	3	4,8%	
	2	23	42,5%	19	30,6%	
	3	7	13,0%	20	32,3%	
	4	4	7,4%	4	6,5%	
	5	3	5,6%	4	6,5%	
	6	0	0,0%	3	4,8%	
	9	0	0,0%	1	1,6%	
preoperative chronic pain	yes	10	18,5%	14	22,6%	0,590
	no	44	81,5%	48	77,4%	
preoperative opioid analgesics	yes	3	5,6%	5	8,1%	0,595
	no	51	94,4%	57	91,9%	
surgical procedure type	partial nephrectomy	24	44,4%	9	14,5%	**< 0,000**
	radical nephrectomy	30	55,6%	53	85,5%	

Bold values indicate *p* < 0.05, which may point to a potentially clinically relevant difference.

### 3.2 Analysis of the main groups (minimally invasive and open)

#### 3.2.1 Outcome parameters

##### 3.2.1.1 Postoperative pain intensity

Table 2 presents the results of the postoperative pain assessment during the first postoperative days. Data was collected on average 1.97 (SD ± 1.22) days postoperatively.

**Table 2:** Postoperative NRS pain scores and satisfaction: minimally invasive vs. open renal surgery.

Variable	Group	n	mean	SD	median	IQR	minimum	maximum	*p*-value
Pain on exercise	minimally invasive	54	5,7	2,19	6	4–8	1	9	
	open	62	6,6	2,19	7	5–8	0	10	**0,050**
	total	116	6,2	2,22	6	5–8	0	10	
Maximum pain	minimally invasive	54	6,6	2,20	7	5–8	2	10	
	open	62	7,8	2,09	8	6–9,8	3	10	**0,004**
	total	116	7,2	2,21	8	6 -9	2	10	
Minimum pain	minimally invasive	54	1,2	1,47	1	0–2	0	5	
	open	62	1,9	1,96	2	0–3	0	6	**0,030**
	total	116	1,59	1,77	1	0–3	0	6	
Satisfaction with pain therapy	minimally invasive	54	7,61	2,390	8	6–10	1	10	
	open	62	7,66	2,127	8	6–9	0	10	0,905
	total	116	7,64	2,243	8	6–10	0	10	

Bold values indicate *p* < 0.05, which may point to a potentially clinically relevant difference.

Patients who underwent minimally invasive procedures (laparoscopic and robot-assisted), pain on exercise reached a median of 6 [IQR 4–8] on the Numerical Rating Scale (NRS). The median maximum pain score was 7 points [IQR 5–8], and minimal pain was reported at a median of 1 [IQR 0–2].

In the open surgery group, pain on exercise reached a median of 7 [IQR 5–8] on the NRS. The median maximum pain score was 8 [IQR 6–9.8], while minimal pain was reported at a median of 2 [IQR 0–3].

Relevant differences between the two groups were observed for maximum pain (*p* = 0.004) and minimal pain (*p* = 0.030), as well as for pain on exercise (*p* = 0.050).

##### 3.2.1.2 Process parameters

Minimally invasive procedures were associated with longer surgical [median: 149.5 min [IQR 125.3–191.5] vs. 98.5 min [IQR 77.3–130.0] min; *p* < 0.001] and anesthesia times [median: 241.5 min [IQR 215.5–286.3] vs. 186 min [IQR 150.8–218.0]; *p* < 0.001].

##### 3.2.1.3 Analgesic consumption after operation

In the post-anesthesia care unit (PACU), approximately the same proportion of patients who underwent minimally invasive surgery vs. open surgery required an opioid [36/51 (70.6%) vs. 45/57 (78.9%); *p* = 0.152]. However, during the further course of their hospital stay, there was an increased need for opioids after open surgery [25/62 (40.3%)] compared to minimally invasive surgery on the ward [11/54 (20.4%); *p* = 0.021]. Particularly after open partial nephrectomy, there was a higher need for both non-opioids in the PACU [minimally invasive: 2/24 (8.3%) vs. open: 5/8 (62.5%); *p* = 0.005] and on the ward [minimally invasive: 15/24 (62.5%) vs. open: 9/9 (100%); *p* = 0.039], as well as for opioids on the ward [minimally invasive: 2/24 (8.3%) vs. open: 5/9 (55.6%); *p* = 0.009].

##### 3.2.1.4 Functional limitations and reported side effects following operation

When considering the overall cohort, pain-related functional impairments most commonly affected coughing and deep breathing (77.6%), as well as movement (75.8%), with no relevant differences observed between the surgical groups. Fatigue was reported by 67.2% of patients across both groups. Differences between the surgical methods were evident for dizziness, which occurred more frequently after minimally invasive procedures [25/54 (46.3%) vs. 16/62 (25.8%); *p* = 0.021].

##### 3.2.1.5 Satisfaction with pain treatment

There were no differences between the groups in terms of satisfaction with postoperative pain management. Patients in both groups rated the treatment with a median score of 8 points [minimally invasive: IQR 6–10; open: IQR 6–9] on a 0–10 rating scale (*p* = 0,905).

##### 3.2.1.6 Extent of resection

In case of nephrectomies, there were no differences in pain intensity between the surgical procedures (pain on exercise: minimally invasive median 7 [IQR 5–8], open 6 [IQR 5–8], *p* = 0.870; maximum pain: minimally invasive median 8 [IQR 6,25–8,75], open 6 [IQR 6–9], *p* = 0.426; minimal pain: minimally invasive median 1 [IQR 0–2], open 2 [IQR 0–3], *p* = 0.208). However, patients who underwent minimally invasive nephrectomies complained more frequently of fatigue [26/30 (86.7%) vs. 31/53 (58.5%); *p* = 0.008] and dizziness [16/30 (53.3%) vs. 13/53 (24.5%); *p* = 0.008]. They also expressed a desire for more intensive pain management more frequently [8/30 (26.7%) vs. 4/53 (7.5%); *p* = 0.024]. Patients in both groups reported substantial interference with movement [minimally invasive: 22/30 (73.3%), open: 40/53 (75.5%), *p* = 0.830] as well as with coughing and deep breathing [minimally invasive: 27/30 (90.0%), open: 39/53 (73.6%), *p* = 0.075]. Opioid analgesics were frequently administered in the post-anesthesia care unit (PACU) in both groups [minimally invasive: 22/29 (75.9%), open: 38/51 (77.6%), *p* = 0.864].

Open surgical partial nephrectomy was associated with the highest pain intensities. The median pain on exercise was 8 [IQR 7–10] compared to 5 [IQR 3–6.3] after minimally invasive procedures (*p* = 0.002). The median maximum pain score was 9 [IQR 8–10] compared to 6 [IQR 4–7.3] after minimally invasive procedures (*p* < 0.0001). Patients in both groups reported substantial interference with movement [minimally invasive: 17/24 (70.8%), open: 9/9 (100%), *p* = 0.149].

#### 3.2.2 Multivariable regression

##### 3.2.2.1 Minimally invasive procedures

Following the identification of potentially clinically relevant variables in univariable analysis (Supplementary Tables S5, S6), potential influencing factors were further examined in multivariable regression models. The comprehensive results are presented in the Supplementary Tables S7–S16.

In summary, within the minimally invasive renal surgery cohort, younger patients (maximum pain: B = −1.34; *p* = 0.019), and patients undergoing nephrectomy (pain on exercise: B = 1.61; *p* = 0.007; maximum pain: B = 1.29; *p* = 0.025) demonstrated a particular vulnerability to high postoperative pain intensity. The presence of comorbidities (B = −1.82; *p* = 0.006) and the absence of an inpatient analgesic prescription (B = 2.21; *p* = 0.006) were associated with reduced patient satisfaction. Regarding pain-related and postoperative symptom complexes, female sex [OR = 0.13 (95% CI 0.03–0.57); *p* = 0.006] and prolonged anesthesia duration [OR = 6.71 (95% CI 1.53–29.52); *p* = 0.012] emerged as relevant subgroups for postoperative nausea, while male sex [OR = 5.08 (95% CI 1.34–19.21); *p* = 0.017] was identified as a risk factor for impaired mood. Sleep disturbances were more frequently observed in younger patients [OR = 7.82 (95% CI 1.86–32.96); *p* = 0.005], and postoperative fatigue appeared to be associated with nephrectomy [OR = 4.64 (95% CI 1.23–17.54); *p* = 0.024].

##### 3.2.2.2 Open procedures

In particular, patients following open partial nephrectomy represent a distinct risk group, characterized by higher pain on exercise (B = −1.92; *p* = 0.006) and maximum pain intensity (B = −1.55; *p* = 0.019), as well as a more frequent desire for additional analgesic treatment [OR = 7.09 (95% CI 1.00–50.33); *p* = 0.050]. Consistent with the findings in the minimally invasive cohort, younger patients (B = −1.54; *p* = 0.002) more frequently reported dissatisfaction. Patients without PONV prophylaxis [OR = 6.03 (95% CI 1.06–34.20); *p* = 0.043] suffered significantly more often from postoperative nausea. The absence of preoperative pain education [OR = 4.59 (95% CI 1.26–16.76); *p* = 0.021] was associated with postoperative mood disturbances. Patients with lower anesthetic risk (ASA I/II) [OR = 4.73 (95% CI 1.16–19.29); *p* = 0.030] more frequently experienced sleep disturbances.

### 3.3 Explorative subgroup analysis according to surgical procedure

#### 3.3.1 Pain intensity, side effects and analgesic consumption

**Figure 2:**
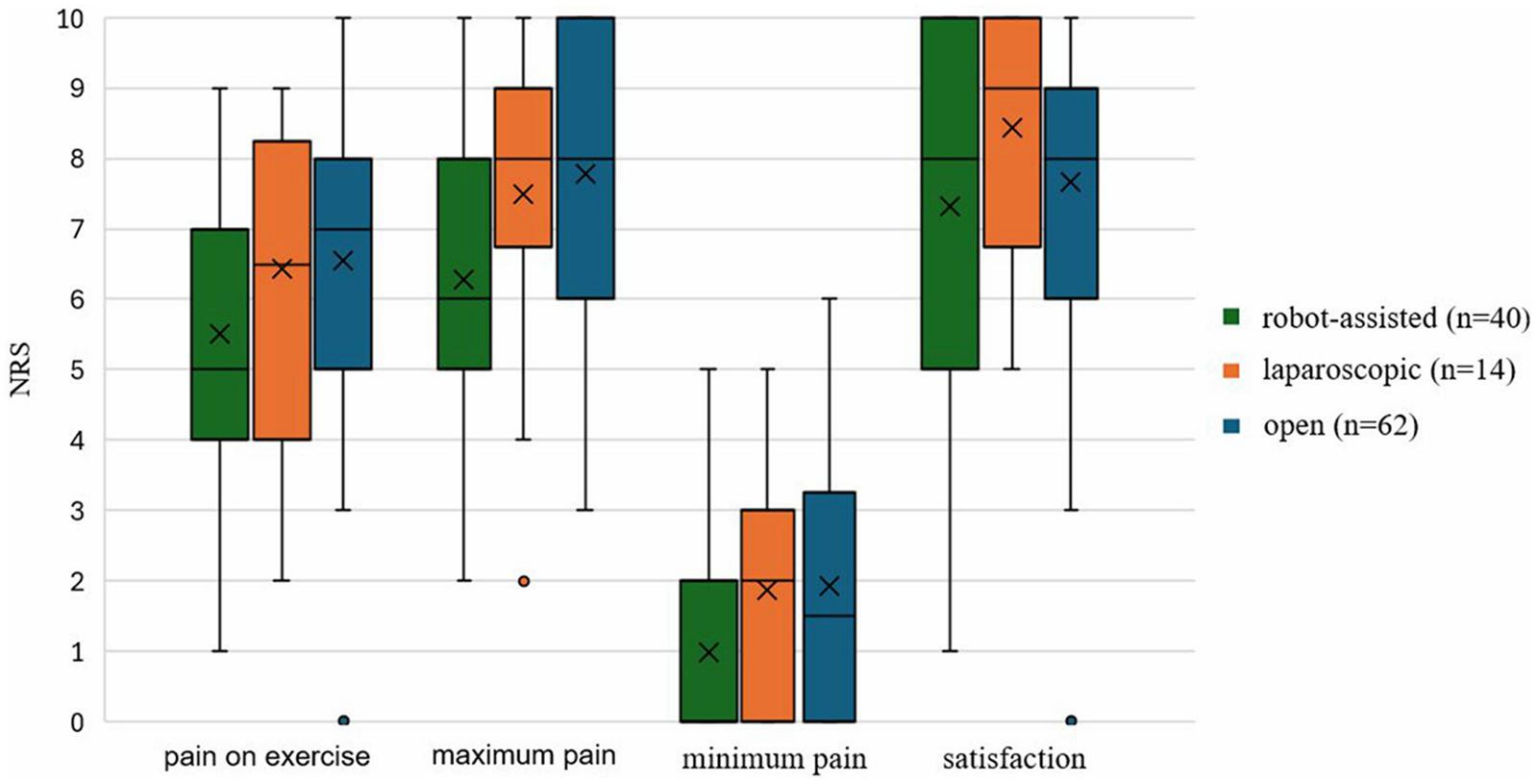
Pain intensity and satisfaction in robot-assisted, laparoscopic, and open renal surgery. Higher values indicate greater levels of pain and satisfaction (see Methods section).

A differentiated analysis of the surgical procedures provided additional evidence of differences between the subgroups (Figure 2). A comparison between robot-assisted and open surgical procedures revealed markedly lower pain scores following robot-assisted procedures. This applied to pain on exercise [median 5 (IQR 4–7) vs. 7 (IQR 5–8); *p* = 0.021], maximum pain [median 6 (IQR 5–8) vs. 8 (IQR 6–9.8); *p* = 0.001], and minimum pain [median 0 (IQR 0–2) vs. 2 (IQR 0–3); *p* = 0.017]. Furthermore, robot-assisted surgery was associated with a reduced requirement for opioid analgesics on the ward [robot-assisted: 6/40 (15.0%) vs. open: 25/62 (40.3%), *p* = 0.005].

When comparing conventional laparoscopic and open surgical procedures, no relevant differences in pain intensity were found. However, patients who underwent conventional laparoscopic surgery reported fatigue [laparoscopic: 13/14 (92.9%) vs. open: 38/62 (61.3%); *p* = 0.028] and dizziness [laparoscopic: 9/14 (64.3%) vs. open: 16/62 (25.8%); *p* = 0.010] more frequently.

Robot-assisted and conventional laparoscopic procedures differed in some respects concerning the outcome parameters. While pain on exercise and satisfaction were comparable, robot-assisted procedures resulted in lower maximum [median 6 [IQR 5–8] vs. 8 [IQR 7–9]; *p* = 0.044] and minimal pain [median 0 [IQR 0–2] vs. 2 [IQR 0.25–3]; *p* = 0.049]. Moreover, a markedly reduced consumption of non-opioid analgesics in the post-anesthesia care unit (PACU) was observed following robot-assisted procedures [robot-assisted: 4/39 (10.25%) vs. conventional laparoscopic: 6/14 (42.85%), *p* = 0.014].

No differences were found between the subgroups in terms of pain-related functional impairment, the need for more intensive pain therapy, or preoperative pain education.

#### 3.3.2 Length of hospital stay

The median length of hospital stay was 7 days [IQR 6.8–9] after robot-assisted surgery and 8 days [IQR 7–9] after conventional laparoscopic surgery. After open surgery, the median length of stay was 10 days [IQR 9–14]; *p* < 0.001) (Table 3).

**Table 3:** Hospital stay (days): robot-assisted vs. conventional laparoscopic vs. open renal surger*y.*

Variable	Group	n	mean	SD	median	IQR	minimum	maximum
hospital stay (days)	robot-assisted	40	8,2	3,3	7	6,8–9	5	24
	laparoscopic	14	8,3	1,9	8	7–9	6	13
	open	62	13,0	7,4	10	9–14	7	47

## 4 Discussion

This observational study evaluated acute postoperative pain following robot-assisted, conventional laparoscopic, and open renal surgery, using standardized QUIPS assessments. The aim was to characterize pain intensity and pain-related functional limitations across different surgical techniques. Furthermore, potential influencing factors were explored. Overall, the findings confirm that postoperative pain is a clinically relevant issue across all procedures, although minimally invasive techniques were associated with lower pain scores and reduced opioid requirements. Nevertheless, pain levels frequently exceeded by far treatment thresholds even after minimally invasive surgery, underscoring the potential need for structured multimodal pain management. These findings indicate that the surgical technique alone may not be sufficient to determine postoperative pain outcomes, and suggest that perioperative analgesic strategies and consistent pain evaluation play a core role.

These results are consistent with previous studies indicating that minimally invasive surgical techniques are somewhat associated with lower levels of postoperative pain compared to open procedures (18–20). Gerbershagen et al. examined a total of 179 surgical procedures regarding postoperative pain. Patients after open nephrectomies (*n* = 158) presented a higher maximum pain level with a mean value of 4.54 on the NRS than after laparoscopic nephrectomies (*n* = 34) with a mean value of 4.24 on the NRS (14). The markedly lower maximum pain scores in this study compared to our results can be explained by the increased use of additional regional analgesia (*n* = 74/158) in open nephrectomies and more frequent administration of opioids (laparoscopic nephrectomy: mean 19 ± 22 mg morphine equivalents; open nephrectomy: mean 35 ± 33 mg morphine equivalents). However, precise treatment regimens are not documented in this study. Overall, our results—as well as those by Gerbershagen et al.—indicate that postoperative pain is often underestimated, even after minimally invasive procedures (14). This can lead to inadequate postoperative pain management and should be given greater consideration in the future.

Interestingly, despite the overall higher pain intensity after open surgery, our analysis of patient satisfaction with postoperative pain management after kidney surgery shows comparable results between the two groups, with a slight advantage in favor of patients who underwent open surgery. Previous studies have shown that patients often report high satisfaction even in the presence of substantial postoperative pain (21). Postoperative satisfaction is multifactorial in nature and is not solely determined by the level of postoperative pain. Pain-specific preoperative information, the perceived effectiveness of pain medication, and the empathy and communication skills of medical staff are also relevant (22, 23).

Regarding pain-related functional impairments, no relevant differences between the surgical methods were found with the exception of a higher incidence of dizziness after laparoscopic procedures. The longer duration of surgery and anesthesia in laparoscopic procedures is considered a possible reason for the increased incidence of postoperative dizziness. This is described in the literature as a typical, albeit mostly harmless, side effect (24). Although this side effect is not serious, it should be considered in postoperative management.

Our results show that opioids were used markedly more often in the open surgery group than after laparoscopic procedures. This is in line with expectations, as more severe pain requires more intensive pain management. In our study, it can be observed that the regular administration of non-steroidal anti-inflammatory drugs (NSAIDs) and other non-opioid analgesics (NOPAs)—which form the basis of multimodal postoperative analgesia—was often lacking. A predominantly demand-oriented regimen was used instead. This may be a relevant factor in the high levels of postoperative pain observed. Several reviews, including those by Moore et al. (25) and Hearn et al. (26), have demonstrated the effectiveness of NSAIDs and other non-opioid analgesics (NOPA) in acute postoperative pain management. The latter examined eight randomized controlled trials on metamizole, which was the most frequently used non-opioid analgesic in the patient group studied. Ultimately, approximately 7 out of 10 patients experienced good pain reduction after a single administration of metamizole. To achieve satisfactory analgesia, regular administration should be carried out depending on the dosage and half-life of the preparation used (27). This enables more regular use of non-opioid analgesics resulting in reduced opioid consumption, minimized opioid-associated side effects, and increased satisfaction (28). Further analgesic approaches, such as neuraxial techniques (e.g., epidural anesthesia), were employed in only a very small number of cases, while ultrasound-guided regional anesthesia was not performed in any patient. This stands in marked contrast to current Enhanced Recovery After Surgery (ERAS) recommendations. Several studies have demonstrated that both neuraxial techniques and ultrasound-guided regional anesthesia (e.g., quadratus lumborum block) not only reduce postoperative pain intensity but also significantly decrease opioid requirements (29, 30).

Our exploratory subanalysis of partial nephrectomy and radical nephrectomies revealed further noteworthy correlations. Open partial nephrectomy was associated with the highest pain on exercise and maximum pain intensities. They were associated with a substantially increased need for non-opioid analgesics in the recovery room and on the ward, as well as increased opioid use during the inpatient stay. Although severe postoperative pain has already been described in the literature (31), the evidence is currently limited due to a lack of comparative reviews or meta-analyses. In their study, Hager et al. analyzed 140 patients undergoing laparoscopic, open transabdominal, or retroperitoneal partial nephrectomy and found no significant differences in cumulative rest or distress-related pain during the first two postoperative days. Only the laparoscopic group did not receive thoracic epidural analgesia or patient-controlled intravenous analgesia, reflecting the assumption of lower pain levels in minimally invasive procedures. After the second postoperative day, pain remained stable in the laparoscopic group, while it continued to rise in the open surgery groups until day seven (29). The results can be cautiously applied to our study. Due to the small number of cases (9 open surgery patients), further investigations with larger case groups are necessary to confirm the trend found.

Although numerous comparative studies examine the oncological outcomes and process parameters after robot-assisted, conventional laparoscopic, and open kidney surgery (32, 33), there is still a clear need for research on postoperative pain progression and health-related quality of life (QoL) after robot-assisted surgery. Particularly studies based on randomized controlled trials are pending. Our data suggests that robotic-assisted kidney surgery might be associated with considerably less postoperative pain compared to the other procedures mentioned. This observation is partly consistent with the results of the ROBOtic-assisted vs. Conventional Open Partial nephrectomy (ROBOCOP) II trial by Sidoti Abate et al. (34). In the aforementioned randomized study, the authors examined 47 patients after open (*n* = 22) and robot-assisted partial nephrectomy (*n* = 25). Using the Kidney Disease Quality of Life-Short Form (KDQOL-SF), pain and physical functioning were assessed at admission, discharge, and 30 and 90 days postoperatively. Although no differences between the groups were found 90 days after the procedure, there were considerable differences in the scores for pain and physical functioning, with a clear advantage for robot-assisted procedures. These results demonstrate that robot-assisted surgical techniques may offer potential advantages not only in terms of oncological safety and perioperative morbidity, but also in the terms of patient-reported postoperative recovery. Grimm et al. conducted a multicenter, randomized, controlled trial including 240 patients with complex renal tumors who underwent either robot-assisted or open partial nephrectomy (17). In line with our findings, the authors reported clear advantages in favor of robot-assisted procedures regarding opioid consumption, pain intensity, and length of hospital stay, as well as better quality of life and comparable complication rates. Based on the mentioned data, our study provides a potential extension by including additional real-life data on robot-assisted and open partial nephrectomies. In addition, it complements data from laparoscopic procedures and results following radical nephrectomies.

Our study also showed that patients undergoing robotic kidney surgery had a shorter median hospital stay of 7 days [IQR 6.8–9] than those undergoing conventional laparoscopic surgery [median 8 days (IQR 7–9)], and open surgery [median 10 days (IQR 9–14)]. Possible reasons for this include lower postoperative pain levels, resulting in faster mobilization and recovery. Moreover, data from literature suggest a lower rate of bleeding events, transfusion frequency and urinary fistula (35–37). This results in faster recovery and mobilization of patients, which shortens the length of stay. A large meta-analysis by Qu et al. including 17.677 patients compared robot-assisted and open partial nephrectomy across multiple clinical and oncological outcomes. For hospital length of stay, five studies with 1.464 patients show a significantly shorter stay after robot-assisted surgery (OR −1.60), suggesting not only clinical benefits but also reduced economic burden (35). Major advantages of robot-assisted surgery were also evident in radical nephrectomies. The differences between laparoscopic and robot-assisted procedures were smaller, which is consistent with our data (38, 39). The combination of lower pain intensity, reduced complication rates, and accelerated mobilization after robot-assisted kidney surgery could contribute to a more efficient use of resources and turn these procedures into a relevant part of fast-track surgery. The median length of hospital stay observed in the present study (7 days following robotic-assisted, 8 days following laparoscopic, and 10 days following open renal surgery) exceeds the values reported in the current literature. In a systematic review and meta-analysis by Kandi et al. published in 2024, mean lengths of stay in the European context were reported as 3.8 days following robotic-assisted, 5.4 days following laparoscopic, and 7.1 days following open partial nephrectomy (40). The prolonged hospital stay observed in our cohort is most likely attributable to historically established institutional practices, which targeted a length of stay of approximately 7 days. This practice was neither based on a formally defined clinical protocol nor driven by economic considerations, but rather reflects the longstanding procedural conventions of the urology department involved. In the context of modern ERAS concepts and the increasing adoption of minimally invasive surgical techniques, a critical reassessment and adjustment of institutional length-of-stay targets appears warranted.

Furthermore, the specific operative access within each surgical group should be considered as an important variable in future investigations. Emerging evidence suggests that, even within the same surgical modality, the choice of approach may have a relevant impact on perioperative outcomes. Several studies have demonstrated significant differences with respect to intraoperative cardiopulmonary parameters, postoperative analgesic requirements, pain intensity, and length of hospital stay depending on the operative access employed (31, 41, 42)

Taken together, these findings provide a clinically relevant basis for the development and prospective evaluation of a standardized procedure-specific perioperative pain management concept in renal surgery.

### 4.1 Limitations

This study is subject to several limitations. It has a monocentric design with a limited number of cases, particularly in the subgroup of open surgical partial nephrectomy and conventional laparoscopic approaches. The small sample size reduces the statistical power and limits the generalizability of the results. Pain was assessed once in the immediate postoperative period. Thus, no conclusions can be drawn for medium-term or long-term pain progression, i.e., the development of chronic postoperative pain. Perioperative analgesia was not completely standardized and varied according to the individual approach of the treating physicians. This heterogeneity may have contributed to variability in analgesic consumption and pain outcomes. The analysis of pain medication was only semi-quantitative, without considering exact dosages and individual drugs within a substance class.

### 4.2 Outlook

Based on the findings of this study, a structured perioperative pain management concept should be developed to improve patient care. In the preoperative setting, this should include consistent and standardized pain education for all patients, with consideration of communication strategies tailored to patients with pre-existing pain conditions. Intraoperatively, consistent PONV prophylaxis and risk-stratified implementation of regional anesthesia techniques are warranted. In the postoperative setting, the establishment of standardized, risk-stratified analgesic protocols is recommended. Furthermore, incorporating a scheduled administration of non-opioid analgesics and, where indicated, regional anesthesia techniques, as well as the definition of a stepwise analgesic escalation scheme is suggested. Successful implementation of these measures will require structured training programs for medical staff. The proposed measures should be evaluated and further optimized in subsequent prospective studies to assess their clinical impact on perioperative outcomes.

## 5 Conclusion

This prospective analysis suggests that postoperative pain following renal surgery may remain a clinically relevant issue despite modern techniques and multimodal approaches. The minimally invasive and open surgery groups differed with respect to patient characteristics, potentially giving rise to distinct risk constellations that may contribute to less favorable postoperative outcomes. Tailoring perioperative management according to individual risk factors may contribute to an improvement in postoperative pain outcomes. Robot-assisted procedures were associated with substantially lower pain intensity, reduced opioid requirements, and shorter hospital stays, potentially indicating directions for further development in kidney surgery.

Open procedures, especially partial nephrectomy, were associated with higher pain scores, suggesting a potentially vulnerable patient group that may benefit from more intensive and structured pain management. Overall, the results indicate a possible need for more consistent implementation of standardized, multimodal analgesia concepts and closer cooperation between surgical and anesthesiologic disciplines, with prospective evaluation of the proposed concept in subsequent studies.

In the long term, robot-assisted procedures could increasingly become the clinical standard, partly due to shorter recovery times and potential economic advantages. However, further multicenter, randomized studies should be conducted to confirm these findings and systematically evaluate long-term patient-oriented outcomes.

## Data Availability

The datasets generated and analyzed during this study are not publicly available due to data protection regulations. However, they may be obtained from the corresponding author upon reasonable request. Access to the source dataset is restricted to employees of the Department of Anesthesiology and Surgical Intensive Care Medicine, University Hospital Halle (Saale), Germany. Data from the QUIPS Project are available upon reasonable request by contacting the study center at quips@med.uni-jena.de.

## References

[1] Deutsche Interdisziplinäre Vereinigung für Schmerztherapie (DIVS) e.V. S3-Leitlinie Behandlung akuter perioperativer und posttraumatischer Schmerzen, AWMF-Registernummer 001-025l (2021). Available online at: https://register.awmf.org/assets/guidelines/001-025l_S3_Behandlung-akuter-perioperativer-posttraumatischer-Schmerzen_2022-11.pdf (Accessed March 26, 2024).

[2] Kehlet H Jensen TS Woolf CJ. Persistent postsurgical pain: risk factors and prevention. Lancet. (2006) 367(9522):1618–25. 10.1016/S0140-6736(06)68700-X 16698416

[3] Meissner W Huygen F Neugebauer EAM Osterbrink J Benhamou D Betteridge N. Management of acute pain in the postoperative setting: the importance of quality indicators. Curr Med Res Opin. (2018) 34(1):187–96. 10.1080/03007995.2017.1391081 29019421

[4] Simanski C Lefering R Paffrath T Riess P Yücel N Maegele M. Die qualität der postoperativen schmerztherapie beeinflusst die krankenhauswahl. Ergebnisse einer anonymen patientenumfrage. Schmerz. (2006) 20(4):327–33. 10.1007/s00482-005-0451-6 16254722

[5] Meißner W. Qualität der schmerztherapie in deutschland—qualitätsmanagement und -sicherung in der akutschmerztherapie. Anasthesiol Intensivmed Notfallmed Schmerzther. (2016) 51(1):50–5. 10.1055/s-0041-101755 26859473

[6] Allegri M Grossi P. Management of postoperative pain: how accurate and successful is our acute pain management? Minerva Anestesiol. (2012) 78(1):1–3. 21750488

[7] Benhamou D Viel E Berti M Brodner G de Andres J Draisci G. Enquête européenne sur la prise en charge de la douleur et de l'analgésie Postopératoires (PATHOS): les résultats français. Ann Fr Anesth Reanim. (2008) 27(9):664–78. 10.1016/j.annfar.2008.07.092 18774676

[8] Meißner W Erlenwein J Stamer U. Organisation der perioperativen schmerztherapie. Anasthesiol Intensivmed Notfallmed Schmerzther. (2018) 53(4):282–94. 10.1055/s-0043-104671 29742787

[9] Freys SM Pogatzki-Zahn E. Akutschmerztherapie in der Operativen Medizin. Berlin, Boston: De Gruyter (2021).

[10] Meißner W Arnold C Baumbach P Dreiling J Göttermann A Komann M. QUIPS Turns 20: 2 decades of quality improvement and health care research in postoperative pain therapy. Anästh Intensivmed 2022 63:235–42. 10.19224/ai2022.235

[11] Chalasani R van de Vel G Shukla PS Zia SUD Mun S Malasevskaia I. A systematic review of surgical outcomes: comparing robotic-assisted partial nephrectomy and open partial nephrectomy in nephron-sparing surgery for renal tumors. Cureus. (2025) 17(2):e79827. 10.7759/cureus.79827 40166509 PMC11955780

[12] Ljungberg B Bensalah K Canfield S Dabestani S Hofmann F Hora M. EAU Guidelines on renal cell carcinoma: 2014 update. Eur Urol. (2015) 67:913–24. 10.1016/j.eururo.2015.01.005 25616710

[13] Zeuschner P Siemer S. Roboter-assistierte chirurgie des nierenzellkarzinoms—heute ein standard? Aktuelle Urol. (2021) 52(5):464–73. 10.1055/a-1493-1557 34107546

[14] Gerbershagen HJ Aduckathil S van Wijck AJM Peelen LM Kalkman CJ Meissner W. Pain intensity on the first day after surgery: a prospective cohort study comparing 179 surgical procedures. Anesthesiology. (2013) 118(4):934–44. 10.1097/ALN.0b013e31828866b3 23392233

[15] Kehlet H Wilkinson RC Fischer HBJ Camu F. PROSPECT: evidence-based, procedure-specific postoperative pain management. Best Pract Res Clin Anaesthesiol. (2007) 21(1):149–59. 10.1016/j.bpa.2006.12.001 17489225

[16] Meissner W Zaslansky R. A survey of postoperative pain treatments and unmet needs. Best Pract Res Clin Anaesthesiol. (2019) 33(3):269–86. 10.1016/j.bpa.2019.10.003 31785713

[17] Grimm MO Bedke J Nyarangi-Dix J Khoder W Foller S Sommerfeld HJ. Open versus robotic-assisted partial nephrectomy in patients with intermediate/high-complexity kidney tumours: final results of the randomised, controlled, open-label, multicentre trial OpeRa. Ann Oncol. (2025) 36(8):988–98. 10.1016/j.annonc.2025.04.005 40250528

[18] Hemal AK Kumar A. A prospective comparison of laparoscopic and robotic radical nephrectomy for T1-2N0M0 renal cell carcinoma (2009).

[19] Dunn MD Portis AJ Shalhav AL Elbahnasy AM Heidorn C McDougall EM. Laparoscopic versus open radical nephrectomy: a 9-year experience. J Urol. (2000) 164(4):1153–9. 10.1016/S0022-5347(05)67131-5 10992356

[20] Weise ES Winfield HN. Laparoscopic partial nephrectomy. J Endourol. (2005) 19(6):634–42. 10.1089/end.2005.19.634 16053351

[21] Córcoles-Jiménez MP Ruiz-García MV Cervera-Monteagudo B Bernal-Celestino R Herreros-Saez ML Flores-Bautista AB. Postoperative pain intensity and patient satisfaction: a multicentre observational study. Appl Nurs Res. (2025) 81:151898. 10.1016/j.apnr.2024.151898 39864886

[22] Carlson J Youngblood R Dalton JA Blau W Lindley C. Is patient satisfaction a legitimate outcome of pain management? J Pain Symptom Manage. (2003) 25(3):264–75. 10.1016/S0885-3924(02)00677-2 12614961

[23] Trinh LN Fortier MA Kain ZN. Primer on adult patient satisfaction in perioperative settings. Perioper Med (Lond). 10.1186/s13741-019-0122-2 PMC6751608 31548883

[24] Chung F Un V Su J. Postoperative symptoms 24 h after ambulatory anaesthesia. Can J Anaesth. (1996) 43(11):1121–7. 10.1007/BF03011838 8922767

[25] Moore RA Derry S Aldington D Wiffen PJ. Adverse events associated with single dose oral analgesics for acute postoperative pain in adults—an overview of cochrane reviews. Cochrane Database Syst Rev. (2015) 2015(10):CD011407. 10.1002/14651858.CD011407.pub2 26461263 PMC6485338

[26] Hearn L Derry S Moore RA. Single dose dipyrone (metamizole) for acute postoperative pain in adults. Cochrane Database Syst Rev. (2016) 4(4):CD011421. 10.1002/14651858.CD011421.pub2 27096578 PMC6540653

[27] Chou R Gordon DB de Leon-Casasola OA Rosenberg JM Bickler S Brennan T. Management of postoperative pain: a clinical practice guideline from the American pain society, the American society of regional anesthesia and pain medicine, and the American society of Anesthesiologists’. Committee on Regional Anesthesia, Executive Committee, and Administrative Council. J Pain. (2016) 17(2):131–57. 10.1016/j.jpain.2015.12.008 26827847

[28] Gupta A Bah M. NSAIDs in the treatment of postoperative pain. Curr Pain Headache Rep. (2016) 20(11):62. 10.1007/s11916-016-0591-7 27841015

[29] Meineke MN Losli MV Sztain JF Swisher MW Abramson WB Martin EI. Robot-assisted laparoscopic nephrectomy: early outcome measures with the implementation of multimodal analgesia and intrathecal morphine via the acute pain service. World J Urol. (2024) 42(1):117. 10.1007/s00345-024-04801-z 38436828 PMC10912429

[30] Kwak KH Baek SI Kim JK Kim TH Yeo J. Analgesic effect of ultrasound-guided preoperative unilateral lateral Quadratus lumborum block for laparoscopic nephrectomy: a randomized, double-blinded, controlled trial. J Pain Res. (2020) 13:1647–54. 10.2147/JPR.S257466 32753940 PMC7342460

[31] Hager B Herzog SA Hager B Sandner-Kiesling A Zigeuner R Pummer K. Comparison of early postoperative pain after partial tumour nephrectomy by flank, transabdominal or laparoscopic access. Br J Pain. (2019) 13(3):177–84. 10.1177/2049463718808542 31308942 PMC6613070

[32] Hoeh B Wenzel M Eckart O Fleisgarten F Garcia CC Köllermann J. Comparison of peri- and intraoperative outcomes of open vs robotic-assisted partial nephrectomy for renal cell carcinoma: a propensity-matched analysis. World J Surg Oncol. (2023) 21(1):189. 10.1186/s12957-023-03061-2 37349748 PMC10286329

[33] Masoumi-Ravandi K Mason RJ Rendon RA. Robotic-assisted laparoscopic partial nephrectomy vs. laparoscopic and open partial nephrectomy a single-site, two-surgeon, retrospective cohort study. Can Urol Assoc J. (2024) 18(8):245–50. 10.5489/cuaj.8585 38587976 PMC11326722

[34] Sidoti Abate MA Menold HS Neuberger M Kirchner M Haney CM Nuhn P. Quality-of-life outcomes of the ROBOtic-assisted versus conventional open partial nephrectomy (ROBOCOP) II trial. BJU Int. (2024) 134(3):434–41. 10.1111/bju.16407 38816992

[35] Bravi CA Rosiello G Mazzone E Minervini A Mari A Di Maida F. The IRON study: investigation of robot-assisted versus open nephron-sparing surgery. Eur Urol Open Sci. (2023) 49:71–7. 10.1016/j.euros.2022.12.017 36874602 PMC9974968

[36] Peyronnet B Seisen T Oger E Vaessen C Grassano Y Benoit T. Comparison of 1800 robotic and open partial nephrectomies for renal tumors. Ann Surg Oncol. (2016) 23(13):4277–83. 10.1245/s10434-016-5411-0 27411552

[37] Qu H Wang K Hu B. Meta-analysis of clinical outcomes of robot-assisted partial nephrectomy and classical open partial nephrectomy. Int J Surg. (2024) 110(10):6268–81. 10.1097/JS9.0000000000001324 38573087 PMC11487007

[38] Crocerossa F Carbonara U Cantiello F Marchioni M Ditonno P Mir MC. Robot-assisted radical nephrectomy: a systematic review and meta-analysis of comparative studies. Eur Urol. (2021) 80(4):428–39. 10.1016/j.eururo.2020.10.034 33218826

[39] Okhawere KE Milky G Razdan S Shih IF Li Y Zuluaga L. One-year healthcare costs after robotic-assisted and laparoscopic partial and radical nephrectomy: a cohort study. BMC Health Serv Res. (2023) 23(1):1099. 10.1186/s12913-023-10111-8 37838666 PMC10576279

[40] Kandi M Richard PO Violette PD Sreekanta A Hanna S Couban R. Length of hospital stay and procedure time after partial nephrectomy or percutaneous thermal ablation A systematic review and meta-analysis. Can Urol Assoc J. (2025) 19(3):E104–13. 10.5489/cuaj.8906 39418494 PMC11879261

[41] Pacini M Lambertini L Wilkinson NR Fox WB Calvo RS Cannoletta D. Robot-Assisted radical prostatectomy: the impact of patient positioning and surgical access on intraoperative anesthesiologic parameters. Urol Pract. (2025) 12(6):779–90. 10.1097/UPJ.0000000000000877 40710761

[42] Lambertini L Pacini M Polverino P Wilkinson NR Calvo RS Cannoletta D. The anesthesiologic impact of single-port robot-assisted partial nephrectomy: a tertiary referral comparative analysis between full-flank transperitoneal, retroperitoneal, and supine lower anterior access (LAA). J Pers Med. (2025) 15(7):306. 10.3390/jpm15070306 40710423 PMC12297866

