# Supplementary material for: QUIPS-based prospective postoperative pain assessment following nephrectomy and partial nephrectomy in robot-assisted, conventional laparoscopic, and open surgical approaches
Source: Front Surg. 2026 Jun 30;13:1782987. doi: 10.3389/fsurg.2026.1782987 (PMC13364973; doi:10.3389/fsurg.2026.1782987)
Supplement: Supplementary file 1 [file Datasheet1.pdf]

Supplementary Material

1 Supplementary Tables

Supplementary Table S1.
Non-opioid analgesics in the Post-Anesthesia Care Unit (PACU)

Patient number	Surgical procedure	Clonidine i.v.	Metamizol i.v.	Paracetamol i.v.	Parecoxib i.v.
1	RAPN				
2	RAPN				
3	RAPN				
4	RAPN				
5	RAPN	X			
6	RAPN		X		
7	RAPN				
8	RAPN	X			
9	RAPN	X			
10	RAPN	X			
11	RAPN				
12	RAPN	X			
13	RAPN				
14	RAPN				
15	RAPN	X			
16	RAPN			X	
17	RAPN	X			
18	RAPN				
19	RAPN	X			
20	RAPN				
21	RAPN				
22	RAPN				
23	RAPN				
24	RAPN				
25	OPN			X	
26	OPN	X	X		
27	OPN	Not available	Not available	Not available	Not available
28	OPN	X	X		
29	OPN		X		
30	OPN				
31	OPN				
32	OPN				X
33	OPN				
34	RAN			X	
35	RAN				

36	RAN				
37	RAN				
38	RAN	X			
39	RAN	X			
40	RAN				
41	RAN	Not available	Not available	Not available	Not available
42	RAN				
43	RAN				
44	RAN				
45	RAN				
46	RAN				
47	RAN			X	
48	RAN				
49	RAN				
50	LN	X		X	
51	LN				
52	LN				
53	LN				
54	LN				
55	LN			X	
56	LN		X		
57	LN				
58	LN				
59	LN		X		
60	LN			X	
61	LN			X	
62	LN	X			
63	LN				
64	ON				
65	ON	X			
66	ON				
67	ON				
68	ON	Not available	Not available	Not available	Not available
69	ON				X
70	ON				
71	ON				
72	ON				
73	ON		X		
74	ON				
75	ON				
76	ON			X	
77	ON				
78	ON				
79	ON				
80	ON				

81	ON			X	
82	ON				
83	ON		X		
84	ON				
85	ON				
86	ON				
87	ON				
88	ON				
89	ON				
90	ON				
91	ON				
92	ON			X	
93	ON			X	
94	ON				
95	ON				
96	ON	X			
97	ON				
98	ON				
99	ON				
100	ON	X			
101	ON	X			
102	ON				
103	ON	Not available	Not available	Not available	Not available
104	ON	X			
105	ON	X		X	
106	ON			X	
107	ON				
108	ON				
109	ON				
110	ON	Not available	Not available	Not available	Not available
111	ON				
112	ON				
113	ON				
114	ON				
115	ON		X		
116	ON	X			

Abbreviations: PACU, Post-Anesthesia Care Unit; i.v., intravenous; RAPN, robot-assisted partial nephrectomy; OPN, open partial nephrectomy; LN, laparoscopic nephrectomy; ON, open nephrectomy; RAN, robot-assisted nephrectomy. **Additional information:** *Not available* indicates that documentation was incomplete.

Supplementary Table S2.
Opioid analgesics in the Post-Anesthesia Care Unit (PACU)

Patient number	Surgical proce-dure	Hydromor-phone i.v. in mg	Oxycodone i.v. in mg	Pethidine i.v. in mg	Piritramide i.v. in mg	Sufentanil i.v. in µg	Patient-controlled analgesia
1	RAPN		8,0				
2	RAPN						
3	RAPN						
4	RAPN		4,0				
5	RAPN						
6	RAPN		4,0				
7	RAPN	0,7					
8	RAPN	0,4					
9	RAPN						
10	RAPN						
11	RAPN		16,0				
12	RAPN	1,2	8,0				
13	RAPN	1,3					
14	RAPN						
15	RAPN						
16	RAPN						
17	RAPN		4,0				
18	RAPN	0,2					
19	RAPN		9,0				
20	RAPN		7,0				
21	RAPN						
22	RAPN						
23	RAPN		8,0				
24	RAPN		1,2				
25	OPN						
26	OPN	1,8					
27	OPN	Not available	Not available	Not available	Not available	Not available	Not available
28	OPN		13,0				
29	OPN	1,6					
30	OPN		16,0				
31	OPN		10,0				
32	OPN		12,0				
33	OPN	0,6	6,0				
34	RAN	0,8					
35	RAN		6,0				
36	RAN		2,0				
37	RAN						

38	RAN	1,6	12,0				
39	RAN		7,0				
40	RAN						
41	RAN	Not available	Not available	Not available	Not available	Not available	Not available
42	RAN	0,8					
43	RAN			50			
44	RAN						
45	RAN		6,0				
46	RAN						
47	RAN		16,0				
48	RAN		8,0				
49	RAN	0,4					
50	LN		9,0				
51	LN		12,0				
52	LN		12,0				
53	LN		13,0				
54	LN		7,0				
55	LN						
56	LN	0,5					
57	LN						
58	LN	0,4					
59	LN	1,1					
60	LN		6,0		14		
61	LN						
62	LN				10		
63	LN		3,0				
64	ON		10,0				
65	ON		3,0				
66	ON						epidural
67	ON	1,3					
68	ON	Not available	Not available	Not available	Not available	Not available	Not available
69	ON	1					
70	ON	0,8					
71	ON		11,0				
72	ON	0,8					
73	ON		17,0				
74	ON	1,3					
75	ON		10,0				
76	ON		8,0				
77	ON						
78	ON						epidural
79	ON						
80	ON	1					

81	ON	1,4					
82	ON		12,0				
83	ON						
84	ON						epidural
85	ON						
86	ON		14,0				
87	ON		10,0				
88	ON						epidural
89	ON	1,6					
90	ON		10,0				
91	ON	0,8					
92	ON		12,0				
93	ON		18,0				
94	ON		8,0				
95	ON		17,0				
96	ON		16,0				
97	ON		12,0				
98	ON						
99	ON		4,0				
100	ON	0,8					
101	ON	0,8					
102	ON						
103	ON	Not available	Not available	Not available	Not available	Not available	Not available
104	ON	0,8					
105	ON		14,0				
106	ON	2					
107	ON		4,0				
108	ON	4,3				10	
109	ON	0,6					
110	ON	Not available	Not available	Not available	Not available	Not available	Not available
111	ON		11,0				
112	ON						
113	ON		12,0				
114	ON						epidural
115	ON		3,0				epidural
116	ON		8,0				

Abbreviations: PACU, Post-Anesthesia Care Unit; i.v., intravenous; mg, milligram; µg, microgram; RAPN, robot-assisted partial nephrectomy; OPN, open partial nephrectomy; LN, laparoscopic nephrectomy; ON, open nephrectomy; RAN, robot-assisted nephrectomy. **Additional information:** *Not available* indicates that documentation was incomplete. Epidural patient-controlled analgesia was performed using sufentanil and ropivacaine.

Supplementary Table S3.
Non-opioid analgesics on the ward

Patient number	Surgical procedure	Clonidine p.o.	Metamizole i.v.	Metamizole p.o.	Paracetamol i.v.
1	RAPN			X	
2	RAPN				
3	RAPN				
4	RAPN		X		
5	RAPN			X	
6	RAPN			X	
7	RAPN				
8	RAPN				
9	RAPN		X		
10	RAPN		X		
11	RAPN				
12	RAPN		X		
13	RAPN				
14	RAPN		X		
15	RAPN				
16	RAPN				X
17	RAPN		X		
18	RAPN		X		
19	RAPN			X	
20	RAPN		X		
21	RAPN		X		
22	RAPN		X		
23	RAPN				
24	RAPN				
25	OPN		X		
26	OPN		X	X	
27	OPN			X	
28	OPN		X		
29	OPN			X	X
30	OPN	X	X		
31	OPN		X		
32	OPN		X		
33	OPN		X		
34	RAN		X		
35	RAN		X		
36	RAN			X	
37	RAN		X		
38	RAN		X	X	
39	RAN		X	X	
40	RAN				
41	RAN		X		

42	RAN				
43	RAN		X		
44	RAN				
45	RAN				
46	RAN		X		
47	RAN		X	X	
48	RAN		X	X	
49	RAN		X	X	
50	LN		X		
51	LN		X		
52	LN			X	
53	LN		X		
54	LN		X		
55	LN		X		
56	LN		X		
57	LN		X		
58	LN		X		
59	LN				
60	LN		X		
61	LN		X		
62	LN		X		
63	LN		X	X	
64	ON				
65	ON		X		
66	ON				
67	ON		X		
68	ON		X		
69	ON		X		
70	ON		X		
71	ON				
72	ON		X		
73	ON		X		
74	ON			X	
75	ON		X		
76	ON				
77	ON				
78	ON		X		
79	ON			X	
80	ON		X		
81	ON			X	
82	ON			X	
83	ON			X	
84	ON				
85	ON		X		

86	ON		X		
87	ON				
88	ON		X		
89	ON		X		
90	ON				
91	ON			X	
92	ON		X		
93	ON		X		
94	ON		X		
95	ON		X	X	
96	ON		X		
97	ON		X		
98	ON				
99	ON		X		
100	ON		X		
101	ON		X		
102	ON		X		
103	ON		X		
104	ON		X		
105	ON			X	
106	ON			X	
107	ON				
108	ON				
109	ON		X		
110	ON		X		
111	ON		X		
112	ON			X	
113	ON		X	X	
114	ON				
115	ON	X	X		
116	ON		X		

Abbreviations: i.v., intravenous; p.o., per os; RAPN, robot-assisted partial nephrectomy; OPN, open partial nephrectomy; LN, laparoscopic nephrectomy; ON, open nephrectomy; RAN, robot-assisted nephrectomy.

Supplementary Table S4.

Opioid analgesics on the ward

Nr.	Surgical procedure	Hydromorphone i.v. in mg	Hydromorphone p.o. in mg	Hydromorphone retard p.o. in mg	Oxycodone retard p.o. in mg	Oxycodone retard p.o. in mg	Piritramide i.v. in mg	Piritramide s.c. in mg	Tapentadol retard in mg	Tilidin/ Naloxon ret. in mg	Tramadol i.v. in mg	Tramadol p.o. in mg	Patient-controlled analgesia
1	RAPN												
2	RAPN												
3	RAPN												
4	RAPN												
5	RAPN												
6	RAPN				80								
7	RAPN												
8	RAPN												
9	RAPN												
10	RAPN												
11	RAPN												
12	RAPN												
13	RAPN												
14	RAPN												
15	RAPN												
16	RAPN												
17	RAPN						7,5						
18	RAPN												
19	RAPN												
20	RAPN												
21	RAPN												
22	RAPN												
23	RAPN												

24	RAPN												
25	OPN												
26	OPN						22,5						
27	OPN					30							
28	OPN						15,0						
29	OPN						7,5						
30	OPN												
31	OPN												
32	OPN						3,0						
33	OPN												
34	RAN												
35	RAN												
36	RAN												
37	RAN												
38	RAN						13,5						
39	RAN									300			
40	RAN												
41	RAN						7,5						
42	RAN												
43	RAN												
44	RAN												
45	RAN												
46	RAN												
47	RAN												
48	RAN												
49	RAN		1,3	20									
50	LN												
51	LN						15,0						
52	LN												
53	LN						7,5						
54	LN						3,0						
55	LN												

56	LN												
57	LN												
58	LN												
59	LN												
60	LN												
61	LN												
62	LN							3					
63	LN						15,0						
64	ON												
65	ON												
66	ON												epidural
67	ON						7,5						
68	ON												
69	ON												
70	ON												
71	ON			4									
72	ON						3,0						
73	ON					20							
74	ON											150	
75	ON												
76	ON												
77	ON												epidural
78	ON												epidural
79	ON												
80	ON						7,5			200			
81	ON												
82	ON												
83	ON							15					
84	ON												epidural
85	ON						7,5						
86	ON												

87	ON												
88	ON												epidural
89	ON												
90	ON												
91	ON									50			
92	ON												
93	ON												
94	ON										100		
95	ON												
96	ON												
97	ON						3,0		200				
98	ON												
99	ON												
100	ON	9,56*											intra-venous
101	ON						7,5						
102	ON										100		
103	ON												
104	ON												
105	ON												
106	ON												
107	ON												
108	ON	2,88*								100			intra-venous
109	ON												
110	ON												
111	ON						10,5						
112	ON												
113	ON												
114	ON												epidural
115	ON												epidural
116	ON												

Abbreviations: i.v., intravenous; p.o., per os; s.c., subcutaneous; mg, milligram; RAPN, robot-assisted partial nephrectomy; OPN, open partial nephrectomy; LN, laparoscopic nephrectomy; ON, open nephrectomy; RAN, robot-assisted nephrectomy. **Additional information:** Epidural patient-controlled analgesia was performed using sufentanil and ropivacaine. *Hydromorphone was used for intravenous patient-controlled analgesia.

Supplementary Table S5.
Univariable analyses – minimally invasive renal surgery

Predictor	Pain intensity & satisfaction (NRS 0–10)				Functional impairments (yes/no)				Symptoms & request (yes/no)			
	Pain on exertion	Maximum pain	Minimum pain	Satis-faction	Movement	Coughing / deep breathing	Sleep	Mood	Fatigue	Nausea	Dizziness	Request for more analgesia
Age (median)	**0.035**	**0.003**	**0.014**	0.130	0.761	**0.018**	**0.001**	**0.001**	0.535	0.083	0.056	0.735
Sex (male/female)	0.101	0.109	0.573	0.059	0.727	0.959	0.857	**0.017**	0.905	**0.010**	0.121	0.147
Anesthesia duration (median)	0.149	0.972	0.449	0.184	0.362	0.735	0.770	0.580	0.535	**0.021**	0.785	0.735
Operative duration (median)	0.106	0.917	0.985	0.065	0.761	0.735	0.770	0.580	1.000	0.785	0.083	0.735
ASA status (I+II / III+IV)	0.302	0.157	0.302	0.501	0.467	0.497	0.815	0.752	0.322	0.236	0.335	0.497
Surgical procedure (PN / RN)	**0.015**	**0.008**	0.458	0.675	0.839	**0.046**	0.132	0.665	**0.018**	0.561	0.246	0.310
PONV prophylaxis (yes/no)	0.813	0.661	0.217	0.355	0.708	0.101	0.707	0.228	1.000	0.273	1.000	0.667
Intraoperative clonidine (yes/no)	0.696	0.630	0.869	0.782	0.382	0.322	1.000	0.687	1.000	1.000	0.431	0.621
Non-opioid analgesics in PACU (yes/no)	0.881	0.172	**0.005**	0.529	0.706	0.098	0.709	0.492	1.000	0.719	1.000	1.000
Opioid analgesics in PACU (yes/no)	**0.043**	0.152	0.288	0.992	1.000	0.301	0.775	0.658	0.336	0.888	0.234	1.000
Co-analgesics in PACU (yes/no)	0.055	0.356	0.660	0.144	0.722	0.244	0.730	0.323	0.263	1.000	0.275	0.421
Non-opioid analgesics on ward (yes/no)	0.819	0.273	0.689	0.106	0.175	0.448	1.000	0.851	0.479	0.106	0.356	1.000
Opioid analgesics on ward (yes/no)	0.572	0.811	0.689	0.198	0.475	1.000	0.293	0.324	0.448	0.733	0.539	1.000
Therapeutic regimen on ward (yes/no)	0.681	0.446	0.970	**0.015**	0.696	0.367	0.703	1.000	1.000	0.244	0.480	0.367
Preoperative pain education (yes/no)	0.667	0.777	0.311	0.161	0.416	0.489	0.064	0.880	0.528	0.420	0.067	0.728
Non-pharmacological measures	0.199	0.311	**0.012**	0.330	0.362	0.735	0.379	0.268	0.214	0.248	**0.014**	**0.018**
Chronic pain (yes/no)	0.848	0.458	0.051	0.656	1.000	0.408	0.707	0.723	1.000	1.000	0.159	1.000
Preoperative opioid use (yes/no)	0.703	0.775	0.251	0.629	0.382	0.322	1.000	0.687	1.000	1.000	0.431	0.621
CCI (= 0 / > 0)	0.283	0.216	0.416	**0.013**	0.515	1.000	0.516	0.945	0.733	0.216	0.210	0.708

Abbreviations: PN = partial nephrectomy; RN = radical nephrectomy; PACU = post-anesthesia care unit; GA = general anesthesia; RA = regional anesthesia; PCA = patient-controlled analgesia; PONV = postoperative nausea and vomiting; CCI = Charlson Comorbidity Index; NRS = Numerical Rating Scale. **Highlighting:** p-values shaded in green and printed in bold indicate statistical significance ($p < 0.05$); values shaded in yellow indicate trends ($0.05 \leq p < 0.10$).

Supplementary Table S6.
Univariable analyses – open renal surgery

Predictor	Pain intensity & satisfaction (NRS 0–10)				Functional impairments (yes/no)				Symptoms & request (yes/no)			
	Pain on exertion	Maximum pain	Minimum pain	Satis-faction	Movement	Coughing / deep breathing	Sleep	Mood	Fatigue	Nausea	Dizziness	Request for more analgesia
Age (median)	0.059	**0.042**	0.571	**0.029**	0.755	**0.038**	0.082	0.607	0.602	0.263	0.562	1.000
Sex (m/f)	0.360	0.104	0.923	0.212	0.984	0.182	0.477	0.621	0.078	0.082	0.631	0.136
Anesthesia duration (median)	0.732	0.606	0.273	0.459	0.119	0.374	0.082	0.607	0.118	1.000	0.562	1.000
Operative duration (median)	**0.041**	**0.007**	0.364	0.807	**0.001**	0.138	0.562	1.000	1.000	0.576	0.562	0.707
ASA status (I+II / III+IV)	0.723	0.791	0.203	0.802	0.514	0.565	**0.028**	0.661	0.085	0.593	0.114	0.705
Surgical procedure (PN / RN)	0.053	**0.045**	0.943	0.822	0.184	0.434	0.683	0.473	0.462	0.106	0.683	**0.012**
Type of anesthesia (GA / GA+RA)	0.101	**0.016**	0.111	0.208	0.191	1.000	1.000	0.132	0.637	0.313	1.000	1.000
PONV prophylaxis (yes/no)	0.201	0.289	0.117	0.528	0.685	1.000	1.000	0.502	0.736	0.064	0.452	0.623
Intraoperative clonidine (yes/no)	0.408	0.056	0.478	0.803	1.000	1.000	**0.049**	0.300	0.151	0.573	0.272	1.000
Non-opioid analgesics in PACU (yes/no)	0.195	0.161	0.669	0.846	0.420	0.727	1.000	0.677	0.743	0.341	**0.048**	**0.024**
Opioid analgesics in PACU (yes/no)	0.613	0.172	0.470	0.094	0.082	1.000	1.000	0.489	1.000	**0.011**	0.485	1.000
Co-analgesics in PACU (yes/no)	0.486	0.285	0.266	0.947	0.646	0.668	1.000	0.467	1.000	0.106	0.218	0.099
Non-opioid analgesics on ward (yes/no)	0.986	0.751	0.673	0.656	0.256	1.000	1.000	0.501	0.339	1.000	1.000	0.335
Opioid analgesics on ward (yes/no)	0.053	**0.005**	0.068	**0.007**	**0.039**	**0.002**	0.746	**0.018**	0.051	0.320	0.132	0.250
PCA on ward (yes/no)	0.935	0.903	0.680	0.783	1.000	0.434	0.219	0.148	1.000	1.000	1.000	0.590
Therapeutic regimen on ward (yes/no)	0.470	0.677	0.581	0.699	0.347	0.388	0.414	**0.016**	0.468	0.681	0.414	0.581
Preoperative pain education (yes/no)	0.769	0.937	0.114	0.174	0.479	**0.025**	0.475	0.053	0.543	0.942	0.060	0.719
Non-pharmacological measures	**0.001**	**0.006**	**0.001**	**0.015**	**0.021**	0.359	0.234	0.165	0.070	0.223	0.234	0.719
Chronic pain (yes/no)	0.096	0.181	0.543	0.229	0.713	1.000	0.488	0.487	0.794	0.202	1.000	0.670
Preoperative opioid use (yes/no)	0.619	0.469	0.170	0.154	1.000	1.000	0.597	0.641	1.000	0.309	0.315	1.000
CCI (= 0 / > 0)	0.496	0.111	0.931	**0.024**	0.186	0.181	1.000	0.710	0.136	0.214	0.414	1.000

Abbreviations: PN = partial nephrectomy; RN = radical nephrectomy; PACU = post-anesthesia care unit; GA = general anesthesia; RA = regional anesthesia; PCA = patient-controlled analgesia; PONV = postoperative nausea and vomiting; CCI = Charlson Comorbidity Index; NRS = Numerical Rating Scale. **Highlighting:** p-values shaded in green and printed in bold indicate statistical significance ($p < 0.05$); values shaded in yellow indicate trends ($0.05 \leq p < 0.10$).

Supplementary Table S7.

Minimally invasive renal surgery: Multiple linear regression – Pain on exertion

Predictor	B	p-value	95% CI
(Constant)	3.27	0.001	1.40 – 5.14
**Surgical procedure (RN vs. PN)**	**1.61**	**0.007**	**0.46 – 2.76**

n = 53; $R^2 = 0.134$; adjusted $R^2 = 0.117$; $F(1, 51) = 7.87$; $p = 0.007$. B = unstandardized regression coefficient. Reference category: Surgical procedure (PN). Bold print indicates significant predictors ($p < 0.05$). PN = partial nephrectomy; RN = radical nephrectomy; CI = confidence interval.

Supplementary Table S8.

Minimally invasive renal surgery: Multiple linear regression – Maximum pain

Predictor	B	p-value	95% CI
**Model 1**			
(Constant)	7.41	< 0.001	6.61 – 8.20
**Age (< vs. ≥ median)**	**–1.63**	**0.005**	**–2.76 – –0.50**
**Model 2**			
(Constant)	5.26	< 0.001	3.25 – 7.27
**Age (< vs. ≥ median)**	**–1.34**	**0.019**	**–2.45 – –0.23**
**Surgical procedure (RN vs. PN)**	**1.29**	**0.025**	**0.17 – 2.41**

n = 54. Model 1: $R^2 = 0.139$; adjusted $R^2 = 0.123$; $F(1, 52) = 8.43$; $p = 0.005$. Model 2: $R^2 = 0.221$; adjusted $R^2 = 0.191$; $F(2, 51) = 7.25$; $p = 0.002$; $\Delta R^2 = 0.082$; $p = 0.025$. B = unstandardized regression coefficient. Reference categories: Age (≥ median); Surgical procedure (PN). Bold print indicates significant predictors. PN = partial nephrectomy; RN = radical nephrectomy; CI = confidence interval.

Supplementary Table S9.

Minimally invasive renal surgery: Multiple linear regression – Minimum pain

Predictor	B	p-value	95% CI
**Model 1**			
(Constant)	0.95	< 0.001	0.53 – 1.37
**Non-opioid analgesics in PACU (yes vs. no)**	**1.45**	**0.004**	**0.48 – 2.42**
**Model 2**			
(Constant)	0.51	0.055	–0.01 – 1.04
**Non-opioid analgesics in PACU (yes vs. no)**	**1.22**	**0.011**	**0.29 – 2.16**
**Non-pharmacological measures (yes vs. no)**	**0.95**	**0.012**	**0.22 – 1.68**

n = 53. Model 1: $R^2 = 0.150$; adjusted $R^2 = 0.133$; $F(1, 51) = 8.99$; $p = 0.004$. Model 2: $R^2 = 0.251$; adjusted $R^2 = 0.221$; $F(2, 50) = 8.39$; $p = 0.001$; $\Delta R^2 = 0.101$; $p = 0.012$. B = unstandardized regression coefficient. Reference categories: Non-opioid analgesics in PACU (no); Non-pharmacological measures (no). PACU = post-anesthesia care unit; CI = confidence interval. Bold print indicates significant predictors.

Supplementary Table S10.
Minimally invasive renal surgery: Multiple linear regression – Satisfaction with pain therapy

Predictor	B	p-value	95% CI
**Model 1**			
(Constant)	5.67	< 0.001	4.17 – 7.17
**Therapeutic regimen on ward (yes vs. no)**	**2.33**	**0.006**	**0.69 – 3.98**
**Model 2**			
(Constant)	7.08	< 0.001	5.36 – 8.81
**Therapeutic regimen on ward (yes vs. no)**	**2.21**	**0.006**	**0.67 – 3.76**
**Charlson Comorbidity Index (0 vs. ≥ 1)**	**–1.82**	**0.006**	**–3.11 – –0.53**

n = 54. Model 1: $R^2 = 0.135$; adjusted $R^2 = 0.118$; $F(1, 52) = 8.10$; $p = 0.006$. Model 2: $R^2 = 0.253$; adjusted $R^2 = 0.224$; $F(2, 51) = 8.64$; $p = 0.001$; $\Delta R^2 = 0.118$; $p = 0.006$. B = unstandardized regression coefficient. Reference categories: Therapeutic regimen on ward (no); Charlson Comorbidity Index (≥ 1). CI = confidence interval. Bold print indicates significant predictors.

Supplementary Table S11.

Minimally invasive renal surgery: Binary logistic regressions – Pain-related impairments and request for more analgesia

Predictor	p-value	OR (95% CI)
**Impairment of coughing / deep breathing** ($\chi^2(3) = 11.16$; $p = 0.011$; Nagelkerke's $R^2 = 0.297$; $n = 53$)		
Age (< vs. ≥ median)	0.102	4.21 (0.75 – 23.53)
Surgical procedure (PN vs. RN)	0.222	0.38 (0.08 – 1.80)
Non-opioid analgesics in PACU †	0.999	< 0.001
**Impairment of sleep** ($\chi^2(2) = 12.85$; $p = 0.002$; Nagelkerke's $R^2 = 0.297$; $n = 54$)		
**Age (< vs. ≥ median)**	**0.005**	**7.82 (1.86 – 32.96)**
Preoperative pain education (yes vs. no)	0.180	0.41 (0.11 – 1.52)
**Impairment of mood** ($\chi^2(2) = 7.86$; $p = 0.020$; Nagelkerke's $R^2 = 0.183$; $n = 54$)		
Age (≥ vs. < median)	0.176	0.44 (0.13 – 1.45)
**Sex (male vs. female)**	**0.017**	**5.08 (1.34 – 19.21)**
**Impairment by nausea** ($\chi^2(2) = 14.38$; $p = 0.001$; Nagelkerke's $R^2 = 0.325$; $n = 54$)		
**Sex (male vs. female)**	**0.006**	**0.13 (0.03 – 0.57)**
**Anesthesia duration (≥ vs. < median)**	**0.012**	**6.71 (1.53 – 29.52)**
**Impairment by fatigue** ($\chi^2(1) = 5.65$; $p = 0.018$; Nagelkerke's $R^2 = 0.146$; $n = 54$)		
**Surgical procedure (RN vs. PN)**	**0.024**	**4.64 (1.23 – 17.54)**
**Impairment by dizziness** ($\chi^2(4) = 10.28$; $p = 0.036$; Nagelkerke's $R^2 = 0.232$; $n = 54$)		
Age (< vs. ≥ median)	0.094	2.92 (0.83 – 10.28)
Operative duration (< vs. ≥ median)	0.533	1.49 (0.43 – 5.17)
Preoperative pain education (yes vs. no)	0.451	0.61 (0.16 – 2.24)
Non-pharmacological measures (no vs. yes)	0.057	0.30 (0.09 – 1.04)
**Request for more analgesia** ($\chi^2(1) = 5.96$; $p = 0.015$; Nagelkerke's $R^2 = 0.164$; $n = 54$)		
**Non-pharmacological measures (yes vs. no)**	**0.029**	**6.25 (1.20 – 32.47)**

OR = odds ratio; CI = confidence interval; PN = partial nephrectomy; RN = radical nephrectomy; PACU = post-anesthesia care unit. Bold print indicates significant predictors ($p < 0.05$). † Unstable estimate due to complete separation; coefficient not interpretable. The reference category is given second within parentheses (comparison group vs. reference).

Supplementary Table S12.
Open renal surgery: Multiple linear regression – Pain on exertion

Predictor	B	p-value	95% CI
**Model 1**			
(Constant)	5.74	< 0.001	5.07 – 6.42
**Non-pharmacological measures (yes vs. no)**	**1.85**	**0.001**	**0.82 – 2.88**
**Model 2**			
(Constant)	9.52	< 0.001	6.95 – 12.08
**Non-pharmacological measures (yes vs. no)**	**2.11**	**< 0.001**	**1.14 – 3.09**
**Surgical procedure (RN vs. PN)**	**–2.10**	**0.003**	**–3.47 – –0.72**
**Model 3**			
(Constant)	8.80	< 0.001	6.21 – 11.40
**Non-pharmacological measures (yes vs. no)**	**2.08**	**< 0.001**	**1.13 – 3.04**
**Surgical procedure (RN vs. PN)**	**–1.92**	**0.006**	**–3.27 – –0.56**
**Opioid analgesics on ward (yes vs. no)**	**0.97**	**0.048**	**0.01 – 1.93**

n = 62. Model 1: $R^2 = 0.178$; adjusted $R^2 = 0.164$; $F(1, 60) = 12.97$; $p = 0.001$. Model 2: $R^2 = 0.289$; adjusted $R^2 = 0.265$; $F(2, 59) = 12.01$; $p < 0.001$; $\Delta R^2 = 0.111$; $p = 0.003$. Model 3: $R^2 = 0.336$; adjusted $R^2 = 0.302$; $F(3, 58) = 9.79$; $p < 0.001$; $\Delta R^2 = 0.047$; $p = 0.048$. B = unstandardized regression coefficient. Reference categories: Non-pharmacological measures (no); Surgical procedure (PN); Opioid analgesics on ward (no). PN = partial nephrectomy; RN = radical nephrectomy; CI = confidence interval. Bold print indicates significant predictors.

Supplementary Table S13.
Open renal surgery: Multiple linear regression – Maximum pain

Predictor	B	p-value	95% CI
**Model 1**			
(Constant)	7.06	< 0.001	6.40 – 7.72
**Non-pharmacological measures (yes vs. no)**	**1.65**	**0.002**	**0.65 – 2.64**
**Model 2**			
(Constant)	6.36	< 0.001	5.60 – 7.12
**Non-pharmacological measures (yes vs. no)**	**1.60**	**0.001**	**0.67 – 2.53**
**Operative duration (≥ vs. < median)**	**1.43**	**0.003**	**0.51 – 2.36**
**Model 3**			
(Constant)	9.44	< 0.001	6.86 – 12.03
**Non-pharmacological measures (yes vs. no)**	**1.82**	**< 0.001**	**0.90 – 2.73**
**Operative duration (≥ vs. < median)**	**1.16**	**0.014**	**0.25 – 2.08**
**Surgical procedure (RN vs. PN)**	**–1.64**	**0.016**	**–2.96 – –0.32**
**Model 4**			
(Constant)	11.43	< 0.001	8.26 – 14.61
**Non-pharmacological measures (yes vs. no)**	**1.72**	**< 0.001**	**0.83 – 2.61**
**Operative duration (≥ vs. < median)**	**0.94**	**0.046**	**0.02 – 1.85**
**Surgical procedure (RN vs. PN)**	**–1.55**	**0.019**	**–2.83 – –0.26**
**Type of anesthesia (GA+RA vs. GA)**	**–1.89**	**0.044**	**–3.72 – –0.05**

n = 62. Model 1: $R^2 = 0.154$; adjusted $R^2 = 0.140$. Model 2: $R^2 = 0.272$; adjusted $R^2 = 0.247$. Model 3: $R^2 = 0.342$; adjusted $R^2 = 0.308$. Model 4: $R^2 = 0.388$; adjusted $R^2 = 0.345$; $F(4, 57) = 9.02$; $p < 0.001$. B = unstandardized regression coefficient. Reference categories: Non-pharmacological measures (no); Operative duration (< median); Surgical procedure (PN); Type of anesthesia (GA). PN = partial nephrectomy; RN = radical nephrectomy; GA = general anesthesia; RA = regional anesthesia; CI = confidence interval. Bold print indicates significant predictors.

Supplementary Table S14.
Open renal surgery: Multiple linear regression – Minimum pain

Predictor	B	p-value	95% CI
**Model 1**			
(Constant)	1.09	< 0.001	0.50 – 1.67
**Non-pharmacological measures (yes vs. no)**	**1.91**	**< 0.001**	**1.03 – 2.80**
**Model 2**			
(Constant)	0.69	0.041	0.03 – 1.35
**Non-pharmacological measures (yes vs. no)**	**1.91**	**< 0.001**	**1.05 – 2.76**
**Opioid analgesics on ward (yes vs. no)**	**0.99**	**0.025**	**0.13 – 1.86**

n = 62. Model 1: $R^2 = 0.238$; adjusted $R^2 = 0.225$; $F(1, 60) = 18.75$; $p < 0.001$. Model 2: $R^2 = 0.301$; adjusted $R^2 = 0.277$; $F(2, 59) = 12.68$; $p < 0.001$; $\Delta R^2 = 0.063$; $p = 0.025$. B = unstandardized regression coefficient. Reference categories: Non-pharmacological measures (no); Opioid analgesics on ward (no). CI = confidence interval. Bold print indicates significant predictors.

Supplementary Table S15.
Open renal surgery: Multiple linear regression – Satisfaction with pain therapy

Predictor	B	p-value	95% CI
**Model 1**			
(Constant)	8.30	< 0.001	7.64 – 8.95
**Opioid analgesics on ward (yes vs. no)**	**–1.58**	**0.003**	**–2.61 – –0.54**
**Model 2**			
(Constant)	9.00	< 0.001	8.21 – 9.80
**Opioid analgesics on ward (yes vs. no)**	**–1.62**	**0.002**	**–2.60 – –0.65**
**Age (< vs. ≥ median)**	**–1.38**	**0.006**	**–2.33 – –0.42**
**Model 3**			
(Constant)	9.53	< 0.001	8.62 – 10.44
**Opioid analgesics on ward (yes vs. no)**	**–1.62**	**0.001**	**–2.57 – –0.67**
**Age (< vs. ≥ median)**	**–1.54**	**0.002**	**–2.48 – –0.60**
**Non-pharmacological measures (yes vs. no)**	**–1.02**	**0.036**	**–1.97 – –0.07**

n = 62. Model 1: $R^2 = 0.135$; adjusted $R^2 = 0.120$; $F(1, 60) = 9.33$; $p = 0.003$. Model 2: $R^2 = 0.241$; adjusted $R^2 = 0.215$; $F(2, 59) = 9.35$; $p < 0.001$; $\Delta R^2 = 0.106$; $p = 0.006$. Model 3: $R^2 = 0.296$; adjusted $R^2 = 0.260$; $F(3, 58) = 8.14$; $p < 0.001$; $\Delta R^2 = 0.056$; $p = 0.036$. B = unstandardized regression coefficient. Reference categories: Opioid analgesics on ward (no); Age (≥ median); Non-pharmacological measures (no). CI = confidence interval. Bold print indicates significant predictors.

Supplementary Table S16.

Open renal surgery: Binary logistic regressions – Pain-related impairments and request for more analgesia

Predictor	p-value	OR (95% CI)
**Impairment of movement** ($\chi^2(5) = 27.70$; $p < 0.001$; Nagelkerke's $R^2 = 0.561$; n = 62)		
**Operative duration (< vs. ≥ median)**	**0.025**	**0.07 (0.01 – 0.71)**
Opioid analgesics in PACU (–1) †	0.060	0.03 (< 0.01 – 1.14)
Opioid analgesics in PACU (no vs. yes)	0.524	0.54 (0.08 – 3.59)
Opioid analgesics on ward (no vs. yes)	0.109	0.16 (0.02 – 1.52)
Non-pharmacological measures (no vs. yes)	0.117	0.22 (0.03 – 1.47)
**Impairment of coughing / deep breathing** ($\chi^2(3) = 19.41$; $p < 0.001$; Nagelkerke's $R^2 = 0.402$; Hosmer-Lemeshow p = 0.426; n = 62)		
**Opioid analgesics on ward (yes vs. no)**	**0.013**	**15.84 (1.79 – 140.15)**
Age (≥ vs. < median)	0.065	0.26 (0.06 – 1.09)
Preoperative pain education (yes vs. no)	0.074	4.03 (0.87 – 18.60)
**Impairment of sleep** ($\chi^2(4) = 15.47$; $p = 0.004$; Nagelkerke's $R^2 = 0.324$; n = 62)		
Age (< vs. ≥ median)	0.207	2.55 (0.60 – 10.89)
Anesthesia duration (< vs. ≥ median)	0.126	3.06 (0.73 – 12.80)
**ASA classification (III/IV vs. I/II)**	**0.030**	**4.73 (1.16 – 19.29)**
**Intraoperative clonidine (no vs. yes)**	**0.036**	**0.06 (0.01 – 0.84)**
**Impairment of mood** ($\chi^2(3) = 19.20$; $p < 0.001$; Nagelkerke's $R^2 = 0.358$; n = 62)		
**Opioid analgesics on ward (no vs. yes)**	**0.024**	**0.23 (0.06 – 0.82)**
Therapeutic regimen on ward †	0.999	< 0.001
**Preoperative pain education (no vs. yes)**	**0.021**	**4.59 (1.26 – 16.76)**
**Impairment by fatigue** ($\chi^2(4) = 12.71$; $p = 0.013$; Nagelkerke's $R^2 = 0.252$; n = 62)		
**Opioid analgesics on ward (no vs. yes)**	**0.043**	**0.28 (0.08 – 0.96)**
Non-pharmacological measures (no vs. yes)	0.077	0.34 (0.10 – 1.13)
Sex (female vs. male)	0.119	2.74 (0.77 – 9.75)
ASA classification (I/II vs. III/IV)	0.247	2.15 (0.59 – 7.83)
**Impairment by nausea** ($\chi^2(3) = 14.81$; $p = 0.002$; Nagelkerke's $R^2 = 0.303$; n = 62)		
**PONV prophylaxis (no vs. yes)**	**0.043**	**6.03 (1.06 – 34.20)**
Opioid analgesics in PACU (no vs. yes)	0.161	0.15 (0.01 – 2.14)
Opioid analgesics in PACU (–1) †	0.999	< 0.001
**Impairment by dizziness** ($\chi^2(2) = 6.00$; $p = 0.050$; Nagelkerke's $R^2 = 0.146$; n = 57)		
Non-opioid analgesics in PACU (no vs. yes)	0.062	0.29 (0.08 – 1.07)
Preoperative pain education (no vs. yes)	0.178	2.50 (0.66 – 9.49)
**Request for more analgesia** ($\chi^2(3) = 11.70$; $p = 0.009$; Nagelkerke's $R^2 = 0.334$; n = 57)		
**Surgical procedure (PN vs. RN)**	**0.050**	**7.09 (1.00 – 50.33)**
Non-opioid analgesics in PACU (no vs. yes)	0.144	0.26 (0.04 – 1.58)
Co-analgesics in PACU (no vs. yes)	0.164	0.24 (0.03 – 1.79)

OR = odds ratio; CI = confidence interval; PACU = post-anesthesia care unit; PN = partial nephrectomy; RN = radical nephrectomy; PONV = postoperative nausea and vomiting; ASA = American Society of Anesthesiologists. Bold print indicates significant predictors ($p < 0.05$). † Unstable estimate due to (quasi-) complete separation; coefficient not interpretable. The reference category is given second within parentheses (comparison group vs. reference).
